# Severity of Airflow Obstruction and Work Loss in a Nationwide Population of Working Age

**DOI:** 10.1038/s41598-018-27999-6

**Published:** 2018-06-26

**Authors:** Sun Hye Shin, Jihwan Park, Juhee Cho, Don D. Sin, Hyun Lee, Hye Yun Park

**Affiliations:** 1 Division of Pulmonary and Critical Care Medicine, Department of Medicine, Samsung Medical Center, Sungkyunkwan University School of Medicine, Seoul, South Korea; 2 Department of Epidemiology, Johns Hopkins University Bloomberg School of Public Health, Baltimore, Maryland USA; 3 Department of Clinical Research Design and Evaluation, SAIHST, Sungkyunkwan University, Seoul, South Korea; 4 Center for Clinical Epidemiology, Samsung Medical Center, Seoul, South Korea; 5 Department of Epidemiology and Welch Center for Prevention, Epidemiology, and Clinical Research, Johns Hopkins University Bloomberg School of Public Health, Baltimore, Maryland USA; 6 Respiratory Division, Department of Medicine, University of British Columbia, Vancouver, British Columbia Canada; 7 Division of Pulmonary Medicine and Allergy, Department of Internal Medicine, Hanyang University College of Medicine, Seoul, South Korea

## Abstract

The impact of COPD severity on labor force participation and work loss is not well known. This study aimed to describe the characteristics of occupations and to evaluate the reason for work loss based on the severity of airflow obstruction (AO). We performed a cross-sectional study using data from the Korean National Health and Nutrition Examination Survey V−VI. We identified 9,901 people aged 40 to 60 years who had normal or AO in spirometry test results. AO was defined as a pre-bronchodilator forced expiratory volume in 1 second/forced vital capacity <70%. AO was present in 7.6% of the subjects, and 81.5%, 82.9%, and 71.6% of subjects with mild, moderate, and severe-to-very severe AO were in the labor force, respectively. Multivariable analyses revealed that severe-to-very severe AO subjects were more likely to have precarious job (adjusted OR = 4.71, 95% CI = 1.70–13.06) and cite health-related problem as the reason for not being in the labor force (adjusted OR = 3.38, 95% CI = 1.03–11.02). Overall, AO was not associated with any significant changes in labor force participation. However, subjects with severe-to-very severe disease were more likely to drop out of the labor force owing to their health-related problems.

## Introduction

Chronic obstructive pulmonary disease (COPD) is a leading cause of morbidity and mortality worldwide that is responsible for substantial economic and social burden^1,2^. As COPD is disease of aging, with the worldwide “greying” of populations, its prevalence as well as the global disease burden measured by disability-adjusted life years are predicted to increase substantially over the coming decades^3^. Underappreciated is the burden of COPD in the working-age population, which is strikingly high^4^. Young individuals with COPD experience a similar degree of dyspnea, exercise impairment and disease progression as older COPD patients, although the severity of airflow limitation is generally less in younger COPD patients^4^. In turn, COPD patients of working age have a lower probability of labor force participation^5–7^ and higher numbers of missed working days^8,9^. Not surprisingly, these patients are forced to retire prematurely^10^, as shown in an international survey that reported that 45% of COPD patients under 65 years of age had work loss due to their heath conditions^11^.

In particular, COPD severity measured by forced expiratory volume in 1 second (FEV_1_) is an important factor affecting the labor force participation rate^5,6^. In one study, increasing severity of COPD was associated with a decreased probability of labor force participation, though the reduction in labor force participation rate did not increase proportionately with COPD severity^5^. While the relative reduction in the labor force participation rate in mild-to-moderate COPD patients was only 3%, it sharply increased to about 15% in severe-to-very severe COPD patients^5^. However, there is a paucity of studies on the detailed characteristics of occupations in COPD patients who remain in the labor force, (in particular, severe-to-very severe COPD patients), compared to those without COPD; similarly, there is little data regarding reasons for work loss in COPD patients who were not in the labor force based on COPD severity. Thus, we used nationally representative data to evaluate reasons for work loss and to describe the characteristics of the occupations in which subjects with airflow obstruction (AO) work based on the severity of airflow limitation.

## Results

### Study Subjects

AO was present in 7.6% (717/9,901) of the study subjects; mild, moderate and severe-to-very severe AO was present in 309 (45.0%), 376 (49.8%), and 32 (5.2%) subjects, respectively. As seen in Table 1, subjects with AO were more likely to be older, male, and former or current smokers compared to subjects with normal spirometry. Subjects with severe-to-very severe AO were less educated, more likely to have lower family income, live in rural area, and have more comorbidities than other groups. Compared to subjects with normal spirometry (75.1% labor force participation), 81.5%, 82.9%, and 71.6% of mild, moderate, and severe-to-very severe AO subejcts were in the labor force, respectively. Type of occupation differed significantly among subjects in the labor force. The proportion of subjects working as manager/professional or office workers was lower in severe-to-very severe AO subjects compared to other groups, while the proportion of subjects working as skilled labor/machine operators was higher in severe-to-very severe AO subjects compared to other groups.

**Table 1:** Distribution of baseline characteristics of the study population according to presence and severity of AO.

	Normal (n = 9,184)	AO (n = 717)			*P*
		Mild (n = 309)	Moderate (n = 376)	Severe-to-very severe (n = 32)	
Age, yr	49.1 (48.9–49.3)	52.7 (52.1–53.3)	52.4 (51.8–53.1)	53.4 (51.5–55.3)	<0.001
Sex					<0.001
Male	46.7 (45.6–47.8)	82.3 (77.3–86.4)	78.6 (73.4–83.0)	62.7 (42.8–79.1)	
Female	53.3 (52.2–54.4)	17.7 (13.6–22.7)	21.4 (17.0–26.6)	37.3 (20.9–57.2)	
Smoking					<0.001
Current smoker	23.6 (22.5–24.7)	42.1 (35.7–48.6)	45.7 (39.7–51.7)	34.0 (17.7–55.4)	
Former smoker	18.6 (17.7–19.6)	38.1 (31.8–44.9)	26.1 (21.2–31.6)	28.1 (14.6–47.1)	
Education level					<0.001
High school or less	68.3 (66.7–69.8)	76.8 (71.0–81.6)	77.7 (72.7–82.1)	93.1 (79.0–98.0)	
>High school	31.7 (30.2–33.3)	23.2 (18.4–29.0)	22.3 (17.9–27.3)	6.9 (2.0–21.0)	
Comorbidity^a^	56.7 (55.4–58.0)	64.1 (57.7–70.0)	75.0 (69.3–79.9)	81.8 (59.9–93.1)	<0.001
Marital status					0.080
Married or living together	88.9 (88.0–89.7)	89.6 (84.3–93.2)	84.3 (79.2–88.4)	80.0 (61.7–90.9)	
Single/separated/divorced/widowed	11.1 (10.3–12.0)	10.4 (6.8–15.7)	15.7 (11.6–20.8)	20.0 (9.1–38.3)	
Urban residence^b^	80.7 (78.0–83.1)	77.4 (70.9–82.8)	74.6 (68.1–80.2)	67.3 (47.0–82.7)	0.024
Family income^c^					0.046
Low	34.3 (32.9–35.8)	32.5 (26.8–38.7)	38.6 (32.7–44.8)	56.3 (36.8–74.1)	
High	65.7 (64.2–67.1)	67.5 (61.3–73.2)	61.4 (55.2–67.3)	43.7 (25.9–63.2)	
Labor force participation					0.003
In the labor force	75.1 (74.0–76.2)	81.5 (76.3–85.7)	82.9 (78.4–86.7)	71.6 (51.0–85.9)	
Not in the labor force	24.9 (23.8–26.0)	18.5 (14.3–23.7)	17.1 (13.3–21.6)	28.4 (14.1–49.0)	
Type of occupation					<0.001
Manager/professional	20.2 (18.9–21.6)	15.4 (11.1–21.1)	10.7 (7.5–15.0)	2.5 (0.3–16.6)	
Office worker	13.3 (12.4–14.3)	10.6 (6.8–16.2)	11.7 (7.8–17.1)	2.4 (0.3–15.8)	
Service/sales worker	24.3 (23.0–25.7)	21.9 (16.7–28.2)	21.6 (16.4–28.0)	20.7 (8.2–43.3)	
Agriculture/fishery worker	8.2 (6.9–9.8)	10.9 (7.4–15.8)	12.1 (8.3–17.4)	12.7 (4.1–32.9)	
Skilled labor/machine operation	21.8 (20.4–23.1)	29.8 (23.3–37.1)	26.9 (21.3–33.4)	52.4 (30.6–73.3)	
Manual laborer	12.2 (11.3–13.1)	11.3 (7.5–16.7)	17.0 (12.5–22.6)	9.4 (1.6–40.0)	
FEV_1_, %predicted	95.3 (95.0–95.5)	88.6 (87.7–89.5)	70.6 (69.7–71.4)	41.4 (39.0–43.9)	<0.001

Values are mean (95% confidence interval) for age and FEV_1_% predicted, and % (95% confidence interval) otherwise.

^a^Comorbid diseases including hypertension, dyslipidemia, diabetes mellitus, stroke, myocardial infarction, angina, cancer, tuberculosis, or asthma were based on self-reports of physician diagnosis and laboratory data. ^b^The urban regions included Seoul, Busan, Daegu, Incheon, Gwangju, Daejeon, and Ulsan, and the rural regions included all other provinces (including Jeju). ^c^Family income levels were classified into upper half and lower half. AO, airflow obstruction.

### Job Status According to Presence and Severity of AO

**Figure 1:**
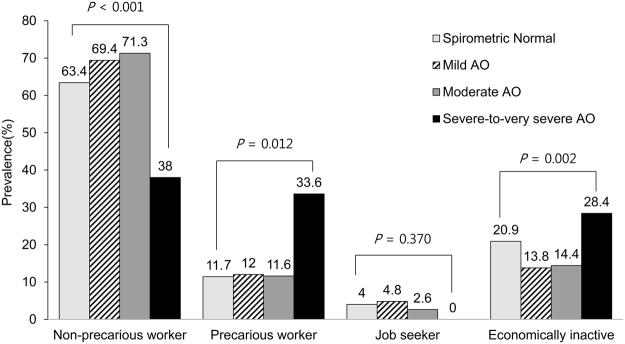
Distribution of job status among working age population in Korea according to the presence and the severity of AO. AO, airflow obstruction.

The proportion of precarious job status was significantly higher in subjects with severe-to-very severe AO (33.6%) than in subjects with normal spirometry (11.7%) or those with mild (12.0%) or moderate (11.6%) AO (P = 0.012) (Fig. 1 and Supplementary Table S1). Among those who were not working, health-related problems were the most common reason in subejcts with AO, and this was most evident in subjects with severe-to-very severe AO (Table 2).

**Table 2:** Reason for not being in the labor force.

	Normal (n = 2,556)	AO (n = 154)			*P*
		Mild (n = 66)	Moderate (n = 80)	Severe-to-very severe (n = 8)	
Health-related problem	25.3 (23.3–27.4)	30.0 (18.7–44.6)	32.8 (21.6–46.4)	73.9 (38.0–92.9)	0.006
Do not need the income	26.3 (24.2–28.5)	21.1 (12.6–33.1)	20.5 (12.4–31.9)	26.1 (7.1–62.0)	0.628
Retired	3.2 (2.5–4.0)	11.3 (4.9–24.1)	11.7 (5.1–24.5)	0 (NA)	<0.001
Laid off	15.9 (14.3–17.7)	25.7 (15.7–39.0)	15.3 (8.0–27.4)	0 (NA)	0.179
Others^a^	29.3 (27.3–31.5)	11.9 (5.1–25.3)	19.7 (11.3–32.0)	0 (NA)	0.009

Values are % (95% confidence interval).

^a^Going to school, Taking care of house or family, or others. AO, airflow obstruction.

### The Impact of AO and Its Severity on Labor Force Participation, Precarious Job Status, and Not Being in the Labor Force Due to Health Problems

As shown in Table 3, there was no significant difference in labor force participation between the subjects after adjustment for age, sex, smoking, comorbidities, education level, and urban residence. However, compared to subjects with normal spirometry, only subjects with severe-to-very severe AO were more likely to have precarious jobs (adjusted odd ratio [OR] = 4.71, 95% confidence interval [CI] = 1.70–13.06) and health-related problems preventing them from being in the labor force (adjusted OR = 3.38, 95% CI = 1.03–11.02).

**Table 3:** The impact of AO and its severity on being in the labor force, precarious job status, and not being in the labor force due to health-related problem.

	Normal (n = 9,184)	AO (n = 717)		
		Mild (n = 309)	Moderate (n = 376)	Severe-to-very severe (n = 32)
In the labor force				
Number (%)^a^	6,628 (75.1)	243 (81.5)	296 (82.9)	24 (71.6)
Unadjusted OR	*Reference*	**1.46** (**1.07–2.00)**	**1.61** (**1.20–2.17)**	0.84 (0.34–2.02)
Adjusted OR^b^	*Reference*	0.76 (0.54–1.07)	0.91 (0.64–1.28)	0.64 (0.19–2.22)
Precarious job status				
Number (%)^a^	1,016 (15.6)	28 (14.8)	41 (14.0)	7 (46.9)
Unadjusted OR	*Reference*	0.94 (0.61–1.46)	0.88 (0.59–1.32)	**4.80** (**1.87–12.34)**
Adjusted OR^b^	*Reference*	1.07 (0.68–1.68)	0.95 (0.62–1.45)	**4.71** (**1.70–13.06)**
Not in the labor force due to health-related problem				
Number (%)^a^	620 (25.3)	18 (30.0)	26 (32.8)	5 (73.9)
Unadjusted OR	*Reference*	1.27 (0.67–2.40)	1.44 (0.81–2.58)	**8.38** (**1.81–38.79)**
Adjusted OR^b^	*Reference*	0.64 (0.32–1.29)	0.67 (0.36–1.26)	**3.38** (**1.03–11.02)**

Values are number (weighted proportions), unadjusted or adjusted OR (95% confidence interval).

^a^With survey weights, participant n may not directly correspond to participant %.

^b^Adjusted for age, sex, smoking, comorbidities, education level, and urban residence. AO, airflow obstruction; OR, odds ratio.

## Discussion

Using KNHANES database, our study showed that prevalence of labor force participation *on average* was not significantly different between subjects with normal spirometry and those with AO, although those with AO were more likely to be employed in precarious jobs. Importantly, we found that health condition was the most common reason for not being in the labor force in subjects with AO, and these findings were particularly pronounced in subjects with severe-to-very severe disease.

In previous studies, the labor force participation rate of individuals with COPD ranged from 56–69%, which was lower than the range of 65–77% seen in those without COPD^5,6,12,13^. Work loss was not linearly associated with COPD severity, but was most prominent in patients with severe disease^5^. In contrast to these findings, we found that there was no significant difference in labor force participation among subjects with AO based on severity of AO. There are several reasons for this phenomenon. The relatively small number in severe AO group in our study might be one reason for this neutral result. Secondly, the overall proportion of labor force participation of AO subjects in our study, which used data from 2010 to 2015, is larger than in previous studies that used data from the mid-1990s to early 2000s. This could be explained in part by improvements in COPD management in the past two decades^14–16^. Finally, there might be gender effect on job status in population with AO. It is well-known that female gender is a major determinant associated with work loss in COPD, and that men have significantly higher (up to 18 times) labor force participation than women independent of COPD^5,17^. Almost 80% of subjects with AO in our study were men, which is higher than reported in previous studies from Western countries^5,6,12,13^. Thus, in our study relatively high proportion of men in the AO group might have diluted the impact of COPD on work loss.

**Figure 2:**
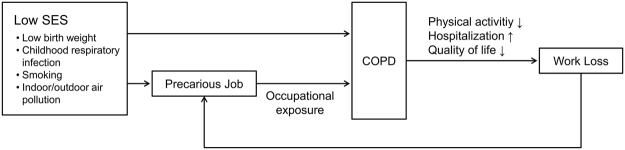
Conceptual diagram of interrelations between COPD and precarious job. SES, socioeconomic status; COPD, chronic obstructive pulmonary disease.

Another important observation in our study is the association between severe-to-very severe AO and precarious job status. Although the labor force participation rate did not differ across severity of airflow limitations, the proportion of subjects working as precarious workers was significantly higher in severe-to-very severe AO. In previous studies using data from the 3^rd^ NHANES, occupations such as freight, stock, material handlers, sales, transportation, machine operators, agriculture, or construction laborers were associated with increased risk of COPD, and the authors claimed that 19.2% of COPD was attributable to occupational exposure^18^. However, precarious jobs and COPD might share a common background. Previous studies showed that there is a strong relationship between COPD prevalence and low socioeconomic status^19,20^, and risk factors such as tobacco use, respiratory tract infections during childhood, and indoor and outdoor air pollution are more prevalent in the disadvantaged population, contributing to COPD development and progression^21–25^. In line with these findings, our study showed that subjects with severe-to-very severe AO are less educated, had lower family income, lived in more rural areas, and had more comorbidities, which might have limited their engagement in high-quality jobs. In addition, chronic and progressive dyspnea has a negative impact on physical activity^26^, and exacerbations result in frequent hospitalizations, impaired outdoor activity, and worsened quality of life^27–29^. A previous retrospective study of severe and very severe COPD patients showed that severity and frequency of recent exacerbations were associated with work productivity impairment^30^. Thus, it might be that subjects with AO, especially severe AO, cannot maintain more stable jobs, but remain in more precarious jobs. Indeed, health problem was the major reason cited for not being in the labor force in this study. This is in agreement with a previous study reporting that 63% of COPD patients who stopped working reported health-related problem as the reason^6^. Thus, it is possible that precarious positions are the result of “partial work loss” due to AO (Fig. 2).

Our study has some limitations. First, despite the significant association between severe-to-very severe AO and precarious job status, the directionality of causation cannot be derived from our study due to its cross-sectional nature. A longitudinal study with detailed outcome measurements is needed to further investigate the impact of COPD on job status. Second, although we were able to tell that some participants were not in the labor force because of health-related problems, we did not have detailed information about the health-related problems. Therefore, work loss that was directly attributable to AO could not be estimated in our study. As previous studies showed that only 26–39% of COPD patients stopped work due to COPD itself, and that comorbidities were associated with work loss in COPD patients^6,9,10^, further studies are necessary to specify comorbidities affecting work loss according to severity of COPD. Finally, since our study used pre-bronchodilator spirometry to define AO, AO group might have included subjects with asthma as well as COPD. However, in this study population aged over 40 years, the weighted prevalence of physician-diagnosed asthma was only 2%, and this group may represent those with overlapping features of asthma and COPD^31^.

In conclusion, our study highlights that the impact of AO on job status differed by severity of AO. While most subjects with mild to moderate AO maintain their work under non-precarious job conditions, a considerable proportion of those with severe-to-very severe AO are precarious workers and cite health-related problem as the major reason for work loss. Thus, more attention from the public and medical communities is warranted for this subset of patients with severe disease.

## Methods

### Study Populations

The Korean National Health and Nutrition Examination Survey (KNHANES) was a cross-sectional, national representative survey of the noninstitutionalized South Korean population conducted by the Korean Ministry of Health and Welfare using a stratified, multistage clustered probability sampling design. Sampling units were defined on the basis of household registries, including geographic area, sex, and age groups^32^.

**Figure 3:**
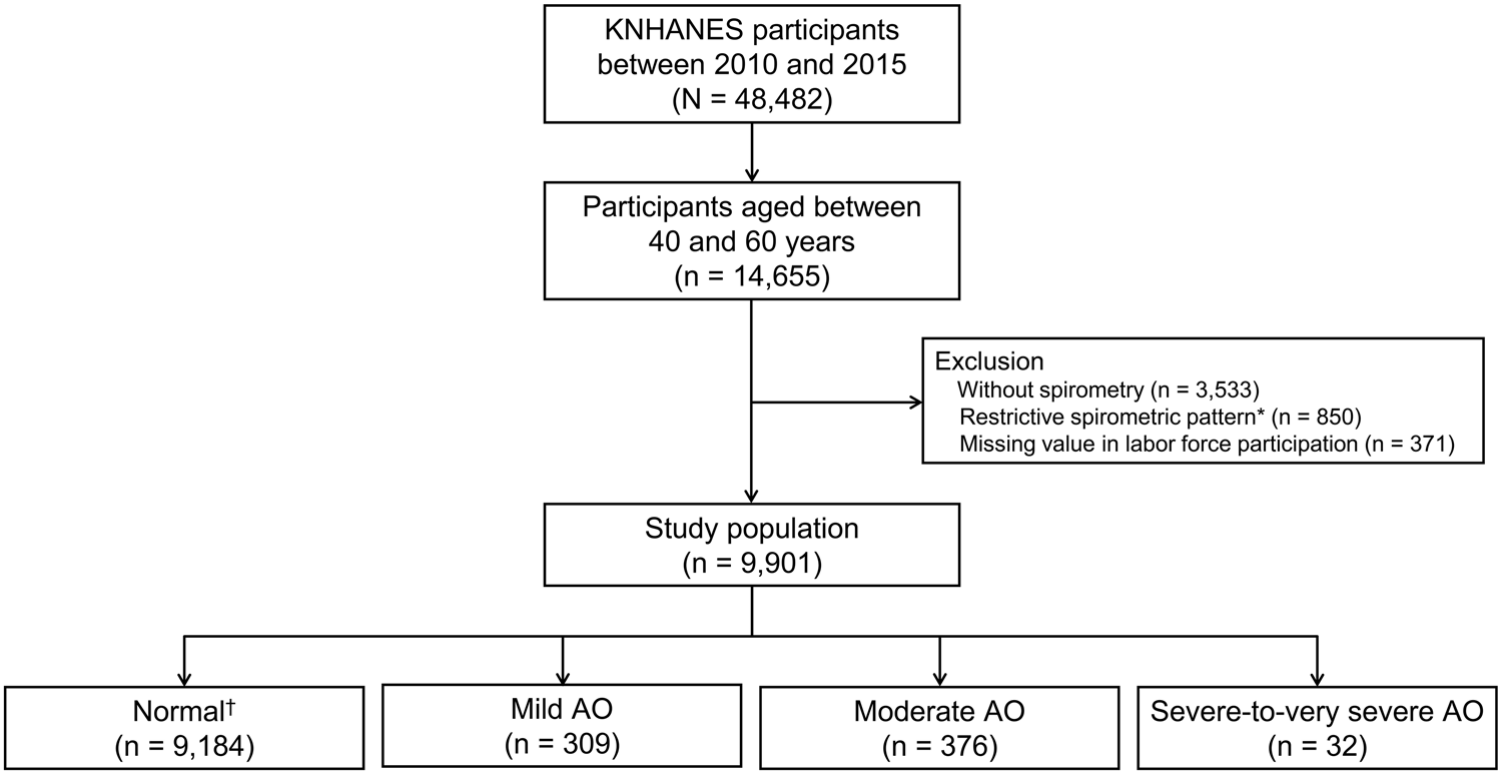
Consort diagram of study population. KNHANES, Korean National Health and Nutritional Examination Survey; AO, airflow obstruction. ^*^Restrictive spirometric pattern was defined as pre-bronchodilator FEV_1_/FVC ≥ 0.7 and FVC < 80% predicted. ^†^Normal in spirometry was defined as pre-bronchodilator FEV_1_/FVC ≥ 0.7 and FVC ≥ 80% predicted.

Information on AO was based on pulmonary function test and it was only available in the 2010 to 2015 survey waves. We restricted our analysis to KNHANES 2010 to 2015 participants between 40 to 60 years of age who were considered to be active in the labor force (N = 14,655). We excluded participants who did not have pulmonary function test (n = 3,533), those with restrictive spirometric pattern without AO (n = 850), and those with missing value in labor force participation (n = 371). Final analysis was based on 9,901 subjects (4,168 men and 5,733 women) (Fig. 3). The 2010–2015 KNHANES study protocols were approved by the Institutional Review Boards of the Korea Centers for Disease Control and Prevention. Written informed consent was obtained from all participants.

### Measurements

KNHANES included a standardized questionnaire administered at home by a trained interviewer and a detailed physical examination at a mobile examination center. All methods were carried out in accordance with the approved guidelines and regulations. Spirometry was performed according to the recommendations of the American Thoracic Society/European Respiratory Society^33^. Absolute values of FEV_1_ and forced vital capacity (FVC) were obtained, and the percentage of predicted values (% predicted) for FEV_1_ and FVC were calculated using the reference equation obtained on analysis of a representative Korean sample^34^. AO was defined as pre-bronchodilator FEV_1_/FVC < 0.70, and severity was classified according to the Global Initiative for Chronic Obstructive Lung Disease (GOLD)^35^ grading system as mild (FEV_1_ ≥ 80% predicted), moderate (50% ≤ FEV_1_ < 80% predicted), or severe-to-very severe (FEV_1_ < 50% predicted). Restrictive pattern was defined as pre-bronchodilator FEV_1_/FVC ≥ 0.70 and FVC < 80% predicted, and normal in spirometry thus defined as pre-bronchodilator FEV_1_/FVC ≥ 0.70 and FVC ≥ 80% predicted.

Being in the labor force was classified according to current working status. Participants were considered to be in the labor force if they worked more than one hour as a paid worker or more than 18 hours as an unpaid family worker for a week^36,37^. Among participants in the labor force, they were classified into two groups based on job security: precarious job status (temporary or daily employees) and non-precarious job status (regular employees, self-employed workers, or unpaid family workers). Type of occupation was asked according to the major groups of the 6th Korean Standard Classification of Occupation and categorized into six groups: managers or professionals, office work, service or sales, agriculture or fishery work, skilled labor or machine operators, and manual laborers^38^. Participants who were on temporary leave of absence were considered as being in the labor force. For people who were not in the labor force, detailed reason for not being in the labor force was asked and categorized into health-related problem, not in need for income, retired, laid off or others (e.g., going to school, taking care of house of family).

Demographic information, education, smoking history, monthly family income and medical history and medication use were determined by self-report. Comorbid condition included hypertension, dyslipidemia, diabetes mellitus, stroke, myocardial infarction, angina, cancer, tuberculosis, and asthma, which were based on self-reports of physician diagnosis and laboratory data.

### Statistical Analysis

All statistical analyses were performed using NHANES weights and svy commands in STATA (version 13; Stata Corp., College Station, TX) to account for the complex multistage probability sampling design. Pulmonary function tested subsample weights were used in all analyses to account for the additional stage of sampling. Multiple logistic regression was used to calculate the (OR) and its 95% CI for the prevalence of being in the labor force, being in the precarious job status, and not-being in the labor force due to health reason and age, sex, smoking status, comorbidities, and education level was adjusted.

## Electronic supplementary material

Supplementary table 1
