# Supplementary material for: Severity of Airflow Obstruction and Work Loss in a Nationwide Population of Working Age
Source: Sci Rep. 2018 Jun 26;8:9674. doi: 10.1038/s41598-018-27999-6 (PMC6018711; doi:10.1038/s41598-018-27999-6)
Supplement: Supplementary file 1 — Supplementary table 1 [file 41598_2018_27999_MOESM1_ESM.docx]

**Severity of Airflow Obstruction and Work Loss in a Nationwide Population of Working Age**

*Sun Hye Shin^1^*^*^*, Jihwan Park^2^*^*^*, Juhee Cho^3,4,5^, Don D. Sin^6^, Hyun Lee^7^*^†^*, Hye Yun Park^1^*^†^

**Supplementary Table S1.** The absolute numbers and weighted proportions of subjects in each job status corresponding to Figure 1.

| Job status | Spirometric Normal^*^  (n=9,184) | AO (n=717) | | | *P* |
| --- | --- | --- | --- | --- | --- |
|  |  | Mild  (n=309) | Moderate  (n=376) | Severe-to-very severe  (n=32) |  |
| Non-precarious worker^a^ | 5,610 (63.4) | 215 (69.4) | 255 (71.3) | 17 (38.0) | <0.001 |
| Precarious worker^b^ | 1,016 (11.7) | 28 (12.0) | 41 (11.6) | 7 (33.6) | 0.012 |
| Job seeker | 386 (4.0) | 18 (4.8) | 13 (2.6) | 0 (0.0) | 0.370 |
| Economically inactive | 2,166 (20.9) | 48 (13.8) | 67 (14.4) | 8 (28.4) | 0.002 |

Values are number (weighted proportions). With survey weights, participant number may not directly correspond to participant %

^a^ Regular employees, self-employed workers, or unpaid family workers

^b^ Temporary or daily employees

^*^ 2 in-labor participants and 4 not-in-labor participants had missing information on job status
